# Lhx2 and Lhx9 Determine Neuronal Differentiation and Compartition in the Caudal Forebrain by Regulating Wnt Signaling

**DOI:** 10.1371/journal.pbio.1001218

**Published:** 2011-12-13

**Authors:** Daniela Peukert, Sabrina Weber, Andrew Lumsden, Steffen Scholpp

**Affiliations:** 1 Karlsruhe Institute of Technology (KIT), Institute of Toxicology and Genetics (ITG), Karlsruhe, Germany; 2 MRC Centre of Developmental Neurobiology, King's College London, United Kingdom; University of Cambridge, United Kingdom

## Abstract

Initial axial patterning of the neural tube into forebrain, midbrain, and hindbrain primordia occurs during gastrulation. After this patterning phase, further diversification within the brain is thought to proceed largely independently in the different primordia. However, mechanisms that maintain the demarcation of brain subdivisions at later stages are poorly understood. In the alar plate of the caudal forebrain there are two principal units, the thalamus and the pretectum, each of which is a developmental compartment. Here we show that proper neuronal differentiation of the thalamus requires Lhx2 and Lhx9 function. In Lhx2/Lhx9-deficient zebrafish embryos the differentiation process is blocked and the dorsally adjacent Wnt positive epithalamus expands into the thalamus. This leads to an upregulation of Wnt signaling in the caudal forebrain. Lack of Lhx2/Lhx9 function as well as increased Wnt signaling alter the expression of the thalamus specific cell adhesion factor *pcdh10b* and lead subsequently to a striking anterior-posterior disorganization of the caudal forebrain. We therefore suggest that after initial neural tube patterning, neurogenesis within a brain compartment influences the integrity of the neuronal progenitor pool and border formation of a neuromeric compartment.

## Author Summary

The thalamus is the interface between the body and the brain. It connects sensory organs with higher brain areas and modulates processes such as sleep, alertness, and consciousness. Our knowledge about the embryonic development of this central relay station is still fragmented. Here, we show that the transcription factors Lhx2 and Lhx9 are essential for the development of the relay thalamus. Zebrafish embryos lacking Lhx2/Lhx9 have stalled neurogenesis - neuronal progenitor cells accumulate but do not complete their differentiation into thalamic neurons. In addition, we find that the neighboring Wnt-expressing epithalamus expands into the space containing mis-specified thalamus in these embryos. We identified a thalamus-specific cell adhesion modulator, Pcdh10b, which is controlled by canonical Wnt signaling. Altered Wnt-dependent Pcdh10b function in Lhx2/Lhx9-deficient embryos leads to intermingling of the thalamus and adjacent brain compartments and consequently regionalization within the caudal forebrain is lost. Organization of the developing CNS into molecularly distinct but transient segments and the implications for regional differentiation are well established for the developing hindbrain. We conclude that this applies to caudal forebrain too: Lhx2 and Lhx9 emerge as crucial factors driving neurogenesis and maintaining the regional integrity of the caudal forebrain. These are two prerequisites for the formation of this important relay station in the brain.

## Introduction

Segmentation is a fundamental step during vertebrate brain development. It involves patterning of the cranial neural tube into distinct and segregated transverse units aligned serially along the longitudinal axis [1]. The most important prerequisite for segmentation are borders between the successive neuromeres to allow individual regionalization, growth, and acquisition of distinct functional identity. This process may be hindered in an embryonic brain by the fact that it rapidly increases in size and complexity. Molecular mechanisms underlying segmentation have been studied during development of the relatively simple hindbrain region [2],[3]. Expression patterns of many regulatory genes also suggest a neuromeric organization of the embryonic forebrain [4],[5]. Recent studies support a segmental forebrain bauplan with three prosomeres (P1–P3) (reviewed in [1]). Based on morphology and gene expression the alar plate of the diencephalon is divided into the prethalamus (P3), thalamus (P2), and pretectum (P1). The epithalamus including epiphysis and habenular nuclei are part of P2. The border between prethalamus and thalamus is defined by compartment borders with the interposed narrow region known as the z*ona limitans intrathalamica* (*ZLI*). Extracellular cell adhesion proteins such as Tenascin within the *ZLI* have been suggested to mediate lineage restriction between the *ZLI* and the anteriorly adjacent prethalamus and posteriorly adjacent thalamus [6]–[8]. Similarly, the *diencephalic-mesencephalic border* (*DMB*), at the posterior limit of the pretectum, has been identified as a compartment boundary where, in addition to Tenascin, an Eph-ephrin dependent mechanism has been suggested to maintain cell segregation [6],[9],[10]. Recent fate mapping studies suggest that the border between the thalamus and the pretectum may also be lineage restricted [11]. However, little is known about a possible mechanism leading to cell lineage restriction between these compartments. The embryonic thalamus (P2) becomes subdivided into two molecularly distinct domains: the rostral thalamus (rTh) marked by expression of the proneural gene Ascl1 and the caudal thalamus (cTh), which expresses Neurog1 [12]–[14]. In tetrapods, the rTh contributes to the majority of the GABAergic neurons in the thalamus including ventral lateral geniculate (vLGN) and intergeniculate leaflet (IGL), whereas the caudal thalamus gives rise to predominately glutamatergic nuclei projecting to the pallium [15]–[17].

LIM homeobox (Lhx) genes regulate developmental processes at multiple levels including tissue patterning, cell fate specification, and growth [18]. These selector genes act as highly similar and highly conserved paralogs. They show a restricted expression pattern in the developing caudal forebrain in frog and mouse; Lhx1/Lhx5 mark the rTh and the pretectum, whereas expression of the Apterous group of Lhx2/Lhx9 is confined to the cTh [19]–[22]. In the mouse, Lhx2 function is required for the acquisition of neuronal identity in different regions such as the telencephalon and nasal placode [23],[24]. In the cortex, Lhx2 is required to limit the adjacent cortical hem, which expresses BMP as well as canonical Wnts. Both signaling pathways orchestrate hippocampal development [25],[26]. This suggests that Lhx2-mediated neurogenesis is involved in maintaining the integrity of cortex. In the diencephalon, the Lhx2/Lhx9 positive cTh is also enriched in Wnt signaling pathway components in monkeys [27]. Correspondingly, this region is located next to sources of canonical Wnt ligands at the *mid-diencephalic organizer* (*MDO*), the signal-generating population in the *ZLI*, and at the diencephalic roof plate [8],[28]. Although the arrangement of these two Wnt positive organizers and the Lhx2/Lhx9 expression pattern in the adjacent Wnt receiving tissue is similar to that in the cortex, our knowledge on their function during diencephalon development is still lacking. During early patterning, Wnt signaling was suggested to have an influence on induction of the thalamus [29]–[31], but the function of Wnts during regionalization remains unclear.

After initial anterior-posterior patterning of the neural tube during gastrulation, it is believed that brain segments develop largely independently. Here we show that Lhx2 and Lhx9 are redundantly required to drive neurogenesis in the zebrafish thalamus. Furthermore, we show that neuronal differentiation mediated by Lhx2/Lhx9 has an impact on maintenance of the thalamus boundaries. Lhx2/Lhx9 restrict the expression of the cell adhesion factor Pcdh10b to the thalamus and therefore sustain the thalamus as a true developmental compartment. Thus, Lhx2/Lhx9 is required for proper development of the thalamus, the core relay station in the brain, and for the integrity of the entire caudal forebrain.

## Results

In zebrafish, the Apterous group of LIM genes contains three members: *lhx2a*, *lhx2b*, and *lhx9* [32]. Lhx2a is expressed only in the early-born olfactory relay neurons [33], whereas Lhx2b resembles the expression pattern of Lhx2 as described in other model organisms. To facilitate species comparison, *Lhx2b* is named as *Lhx2* throughout the article.

### Fine Mapping of the Temporal and Spatial Expression of Lhx2 and Lhx9 in the Caudal Diencephalon

**Figure 1:**
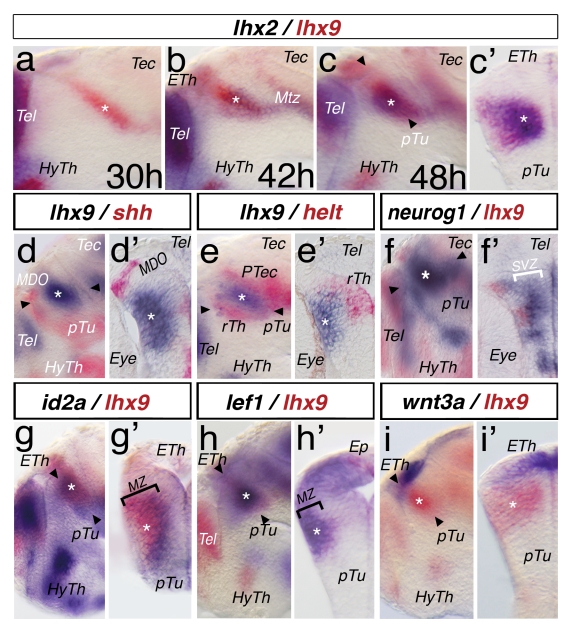
Dynamic expression pattern of *lhx2* and *lhx9* during regionalization of the caudal forebrain. A double in situ hybridization approach for thalamic development. Embryos were mounted laterally (a, b, c, etc.) or sectioned and the left hemisphere is shown (c′, d′, e′, etc.). Plane of section is indicated in the previous picture with black arrowheads. Asterisks mark the position of the thalamus. Marker genes and stages are indicated (a, b), all other embryos (c–i′) are 48 hpf. *lhx2* expression is stained in red and *lhx9* is stained in blue. *lhx9* expression is revealed in the thalamus at 30 hpf (a). At 42 hpf, *lhx9* expression increases and *lhx2* expression is detectable ventro-posteriorly within the *lhx9* domain (b). At 48 hpf, *lhx2* and *lhx9* overlap in the Th (c) and cross-section analysis reveals an overlap of both markers within the mantle zone of the thalamus (c′). The *shh*-positive mid-diencephalic organizer (*MDO*) is located anterior to the *lhx9* positive thalamus (d), and a cryo-section reveals a gap between both expression domains (d′). Helt expression in the rostral thalamus (rTh) and pretectum (PTec) abuts the *lhx9* expression (e, e′). *neurog1* marks the thalamic territory (f) and cross-section in (f′) shows that *neurog1* marks the subventricular zone (SVZ; white bar) and does not overlap with the expression domain of *lhx9* in the mantle zone. The thalamus expression domain of *lhx9* overlaps with the pattern of *id2a* in the medial part of the mantle zone (g, g′, black bar). *lef1* as a marker of post-mitotic thalamic neurons shows co-expression with *lhx9* in the MZ (i, i′; black bar). Notably, *lhx9* expression is seen also in the epiphysis (Ep). The thalamic *lhx9* expression domain abuts the *wnt3a* expression domain in the epithalamus (ETh, g, g′). ETh, epithalamus; HyTh, hypothalamus; Mtz; marginal tecal zone; pTu, posterior tuberculum; Tec, tectum; Tel, telencephalon.

To explore neuronal differentiation in the thalamus, we examined the expression dynamics of *lhx2* and *lhx9* at early stages of caudal forebrain development (Figures 1 and S1). We detect expression of *lhx9* in the diencephalon first at 30 hpf (primordial stage 15; Figure 1a, asterisk), while at 42 hpf (high-pec stage), the *lhx9* expression domain broadens and an overlapping domain of *lhx2* expression becomes apparent (Figure 1b). At 48 hpf (long-pec stage), *lhx2* and *lhx9* are co-expressed in the thalamus (Figure 1c, asterisk). This expression is maintained at later stages (Figure S1). A cross-section validates the overlap of Lhx2 and Lhx9 positive cells, predominantly laterally in thalamic neuroepithilium (Figure 1c′).

At 48 hpf, *lhx9* expression is in proximity to, but with a distinct separation from, the Shh-positive *MDO* and basal plate (Figure 1d,d′). In order to determine the fate of cells in this *shh* and *lhx9 negative domain*, we cloned the zebrafish homolog of the *hey-like transcription factor* (*helt)*. Helt has been described as a specific marker of the prospective GABA interneurons of the rostral thalamus (rTh), pretectum, and midbrain [34],[35] and is required for the formation of these interneurons in the mouse mesencephalon [36]. The expression domain of *helt* abuts the rostral, ventral, and caudal extent of the *lhx9* expression domain (Figure 1e,e′). Complementary to the *helt* expression, we find an overlap with glutamatergic neurons marked by *vglut2.2* at 3 dpf (Figure S1). This suggests that *lhx9* marks the caudal thalamus (cTh) and is absent in the GABAergic rTh and pretectum in zebrafish. The ßHLH factor *neurogenin1* is strongly expressed in an intermediate layer of the neuroepithelium of the cTh, most likely the subventricular zone (Figure 1f,f′). Expression of *neurog1* abuts the expression of *lhx9* in the cTh. The medial part of the *lhx9* expression domain overlaps with the expression of the differentiation marker *id2a* (Figure 1g,g′). The expression domain of the thalamus-specific post-mitotic neuronal marker *lef1* [16],[37] overlaps entirely with *lhx9* (Figure 1h,h′). The dorsal limit of the Lhx9 domain is adjacent to that of Wnt3a, a marker of the central epithalamus (Figure 1i,i′). Nevertheless, the *lhx9* expression domain overlaps with the expression of the Wnt target *axin2* in the diencephalic alar plate (Figure S1), suggesting that Wnt expression at the epithalamus/*MDO* might be required to activate the Wnt signaling cascade in the thalamic territory.

Thus, we can define Lhx2/Lhx9 as a marker for post-mitotic neurons of the thalamic mantle zone in zebrafish at 48 hpf.

### Incomplete Development of Thalamic Neurons in Lhx2 and Lhx9-Deficient Embryos

**Figure 2:**
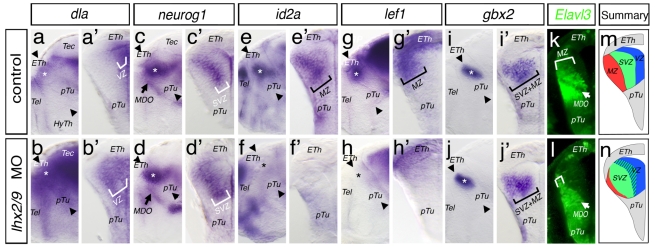
Differentiation of thalamic neurons is stalled in *lhx2/lhx9* morphant embryos. Analysis of embryos for neuronal differentiation in double morphant embryos at 48 hpf, lateral view (a, b, c, etc.), and cross-section of left hemispheres (a′, b′, c′ etc.) of the same embryo are shown. The expression domain of the neuronal precursor *deltaA* at the ventricular zone (VZ) is vigorously expanded in double morphant embryos (a–b′). Expression of the progenitor marker *neurog1* marking the subventricular zone (SVZ, white bars) is also broadened in *lhx2/lhx9* morphant embryos compared to control embryos (c–d′). However, the thalamus-specific terminal differentiation markers, *id2a* and *lef1*, are down-regulated in the mantle zone of the cTh (MZ, black bars) of Lhx2/Lhx9-deficient embryos (e–h′). The postmitotic marker *gbx2* shows no alteration in the compound morphant embryos (i–j′). The number of cells expressing the pan-postmitotic neuronal marker *elavl3* is strongly decreased in the double morphant embryos shown by a confocal microscope section of an transgenic Elavl3:GFP transgenic embryos (k, l). The *deltaA* and *neurog1* positive precursor pool in the ventricular/subventricular zone (blue and green domain) expands on the expense of the post-mitotic thalamic neurons (red domain) in the mantle zone in *lhx2/lhx9* morphant embryos (m, n). ETh, epithalamus; HyTh, hypothalamus; *MDO*, mid-diencephalic organizer; MZ, mantle zone; pTu, posterior tuberculum; VZ, ventricular zone.

At 48 hpf key markers for neurogenesis in the zebrafish brain are expressed in a pattern representing best comparability with amniote brains [38]. Therefore, we chose this stage for the following analyses. To address the function of Lhx2 and Lhx9 in the developing caudal thalamus, we used an antisense Morpholino-based knock-down strategy (Figure S2). Neither *lhx2*−/− zebrafish mutant embryos (bel^tv24^) [39] (*n* = 13) nor single morphant embryos for either *lhx2* or *lhx9* (*n* = 29) are visibly distinguishable from uninjected wild type embryos (Figure S2) similar to the situation in the Lhx2 knock-out mouse. However, *lhx2/lhx9* double morphant embryos showed significant disruption of thalamic structure (Figure 2). This is consistent with their overlapping expression domains in the diencephalon (Figure 1) and suggests a functional redundancy within the Apterous group during caudal thalamus development. Therefore, we focused on an approach to reduce both Lhx2 and Lhx9 messages simultaneously by generating double morphant embryos. In addition, we analyzed the *lhx9* knock-down morphant in the zebrafish *lhx2* mutant background. To define the step in thalamic neuronal differentiation that is dependent on Lhx2/Lhx9 function, we analyzed the expression of the following set of thalamus-specific markers: the neurogenic marker *deltaA* [40], the ßHLH factor *neurog1*, marking early thalamic progenitors [41], a regulator of neuronal differentiation *id2a*, and a marker for mature thalamic neurons *lef1* [16],[42], the caudal thalamus-specific homeobox gene *gbx2* [43],[44], and the pan-neuronal marker *elav-like 3* (formerly Hu antigen C) [45]. These markers can be allocated to three layers in a neuroepithelium in zebrafish: the ventricular proliferation zone (VZ) is positive for *deltaA*, the intermediate or subventricular zone (SVZ) zone is marked by *neurog1*, and the post-mitotic mantle zone (MZ) by *elavl3* [38].

At 48 hpf, we observe a lateral expansion of the *deltaA* positive ventricular zone in *lhx2/lhx9* morphant embryos (36/54; Figure 2a–b′). Likewise, the expression of the proneural factor *neurog1* (*n* = 18) in the subventricular zone expands laterally (Figure 2c–d′). Consequently, the expression of the post-mitotic thalamic neuronal markers *id2a* (19/31) and *lef1* (13/20) is significantly reduced (Figure 2e–h). Interestingly, the Shh-dependent homeobox transcription factor *gbx2* (*n* = 25) as well as the Wnt mediator *tcf7l2* show no alteration in compound morphant embryos (Figures 2i,j′, S3). The pan-neuronal marker *elavl3* is decreased in the mantle zone (3/5; Figure 2k,l). This suggests that DeltaA and Neurog1 positive thalamic progenitors need Lhx2/Lhx9 function to proceed with neuronal differentiation (Figure 2m,n).

**Figure 3:**
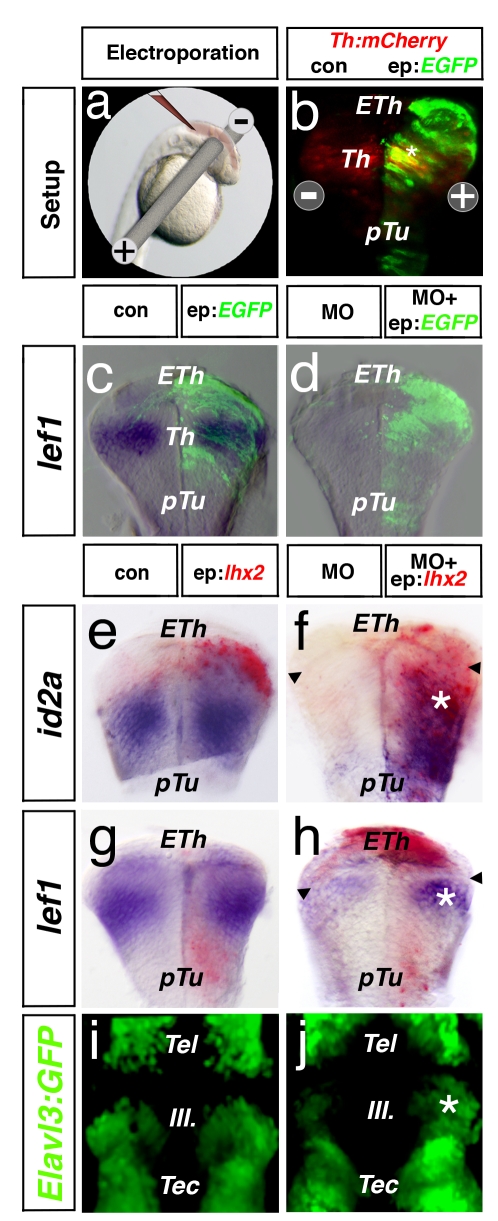
Lhx2 promotes thalamic neurogenesis. At 24 hpf, DNA (indicated in red) was injected into the brain ventricle followed by electroporation approach (a). To validate the specificity and efficiency, we targeted one hemisphere of the thalamus territory with *EGFP* DNA at 24 hpf. We find a co-localization with the thalamus-specific marker *barhl2*:mCherry at 48 hpf (b). Analysis of cross-sections reveal that electroporation of *EGFP* DNA does not alter the expression of *lef1* in wt embryos (c). Furthermore, we find strong down-regulation of *lef1* in *lhx2/lhx9* morphant embryos, which is not altered by *EGFP* DNA electroporation (d). After electroporation of *lhx2* DNA, we observe an unaltered expression of *id2a*, *lef1*, and Elavl3-GFP expression within the endogenous expression site in the electroporated hemispheres (e, g, i). Electroporated side was identified by an ISH against *lhx2* mRNA in red. However, electroporation of *lhx2* DNA at 24 hpf can restore the expression of *id2a*, *lef1*, and Elavl3-GFP in Lhx2/Lhx9-deficient embryos (f, g, j; asterisk). Notably, electroporation of Lhx2 can ectopically induce *id2a* expression in the basal plate—that is, in the pTu (f). pTu, posterior tuberculum; RP, roof plate, Tec, tectum; Tel, telencephalon.

To validate our knock-down strategy and to restrict our analysis temporally and spatially to the thalamus after 24 hpf, we adapted the electroporation technique to the zebrafish system. We were thereby able to deliver DNA unilaterally into the neural tube by pulsed electric stimulation at 24 hpf (Figure 3a) and analyze the thalamus at 48 hpf (Figure 3b). Electroporation of EGFP DNA leads to neither molecular nor morphological alteration of the forebrain/midbrain area (Figure 3c,d; *n* = 15). Based on previous experiments, we asked if Lhx2 function is sufficient for the induction of post-mitotic thalamic neurons in the Lhx2/Lhx9-double-deficient embryos. Therefore, we re-introduced Lhx2 function unilaterally in the thalamus of Lhx2/lhx9 morphant embryos at 24 hpf corresponding to the endogenous onset of Lhx2 expression (Figure 1). At 48 hpf, the loss of *id2a* (7/19), *lef1* (3/15), and *Elavl3:GFP* (8/15) expression within the thalamus of *lhx2/lhx9* morphant embryos was restored in the electroporated hemisphere at 48 hpf (Figure 3f,h,j). It seems that the laterally expanded epithalamus of morphant embryos can be restored in the electroporated hemisphere (arrowheads).

Therefore, we conclude that Lhx2/Lhx9 function is crucial for neurogenesis in the caudal thalamus. Furthermore, Lhx2 alone can compensate for the loss of Lhx2 and Lhx9, suggesting a redundant function between these paralogs during thalamic neurogenesis. Finally, local electroporation is a valid tool to validate the specificity of a knock-down approach in zebrafish.

### Thalamic Neurogenesis Is Required to Limit the *MDO* and Epithalamus

**Figure 4:**
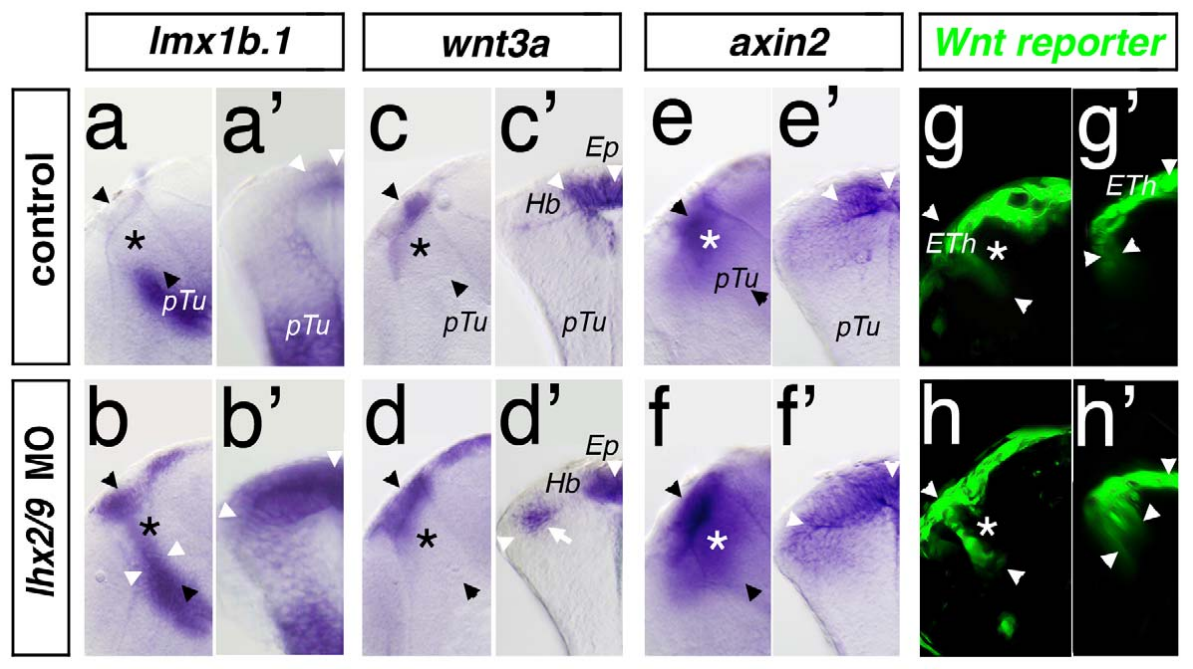
Knock-down of Lhx2/Lhx9 leads to an expansion of the Wnt positive epithalamus. A lateral view (a, b, c, etc.) and a cross-section (a′, b′, c′, etc.) of the left hemisphere of the same embryo at 48 hpf are displayed. Thalamus is marked by asterisks. Section plane of the cross-section is indicated by black arrowheads. In control MO injected embryos, *lmx1b.1* expression domain marks the *MDO* and the dorsal RP (a, a′). Knock-down of Lhx2/Lhx9 leads to an expansion of both areas into the thalamic territory (b, b′). *wnt3a* marks the epiphysis but not the habenula territory (c, c′). In Lhx2/Lhx9-deficient embryos, *wnt3a* expression is ectopically activated in the dorsal part of the thalamus (d, d′). Subsequently the expression of Wnt target genes such as *axin2* (e, e′) as well as the Wnt reporter line 7×TCF-Xla Siam:GFP ia4 (g, g′) shows an expanded expression domain in compound morphant embryos (f, f′ and h, h′). Ep, epiphysis; Hb, habenula; pTu, posterior tuberculum.

In the next set of experiments we analyzed the consequence of Lhx2/Lhx9 deficiency on adjacent tissues: the *mid-diencephalic organizer* (*MDO*) and the embryonic epithalamus (ETh). We find that in morphant embryos the expression domain of *lmx1b.1*, a marker for the *MDO* and the Eth, expands ventro-posteriorly into the thalamus at 36 hpf (31/36; Figure 4a–b′). Similarly, the expression domains of *wnt3a* (89/141) and *wnt1* (8/11) also expand (Figures 4c–d′, S3). A cross-section reveals that the *wnt3a* expression is induced ectopically lateral to the habenula, presumably in the thalamic territory (Figure 4d′, arrow) although the forming habenula remains *wnt3a* negative [46]. To test whether the expanded Wnt expression affects thalamic development, we first monitored Wnt activity in the diencephalon. Here, we analyzed the expression pattern of the pan-canonical Wnt target gene *axin2* at 24 hpf, 48 hpf, and at 72 hpf. As expected, we were not able to detect expansion of *axin2* expression prior to onset of Lhx2/Lhx9 expression in the thalamus (Figure S3). From 48hpf, *axin2* expression is progressively increased in the thalamus of Lhx2/Lhx9-deficient embryos (35/53; Figures 4e–f′, S3). We confirmed these results using a Wnt reporter zebrafish line 7×TCFsiam:GFP, which expresses GFP under the control of seven repetitive TCF-responsive elements driving a minimal promoter. The GFP expression is detectable around known canonical Wnt sources in the diencephalon—that is, the *MDO*/ETh area (Figure 4g,h). Lhx2/Lhx9 morphant embryos show expanded GFP expression in the thalamus (23/35; Figure 4g′,h′). In summary, we find that the knock-down of Lhx2/Lhx9 in zebrafish embryos results in an expansion of the epithalamic expression domain of Wnt ligands. This leads to an enhancement of Wnt signaling in the diencephalon, predominantly in the subjacent thalamus.

### Protocadherin10b Is a Thalamus-Specific Wnt Target

To address the consequences of the loss of Lhx2/Lhx9 and the subsequent upregulation of Wnt signaling on the integrity of the caudal diencephalon, we analyzed the expression pattern of regionally expressed cell adhesion factors in the caudal forebrain.

**Figure 5:**
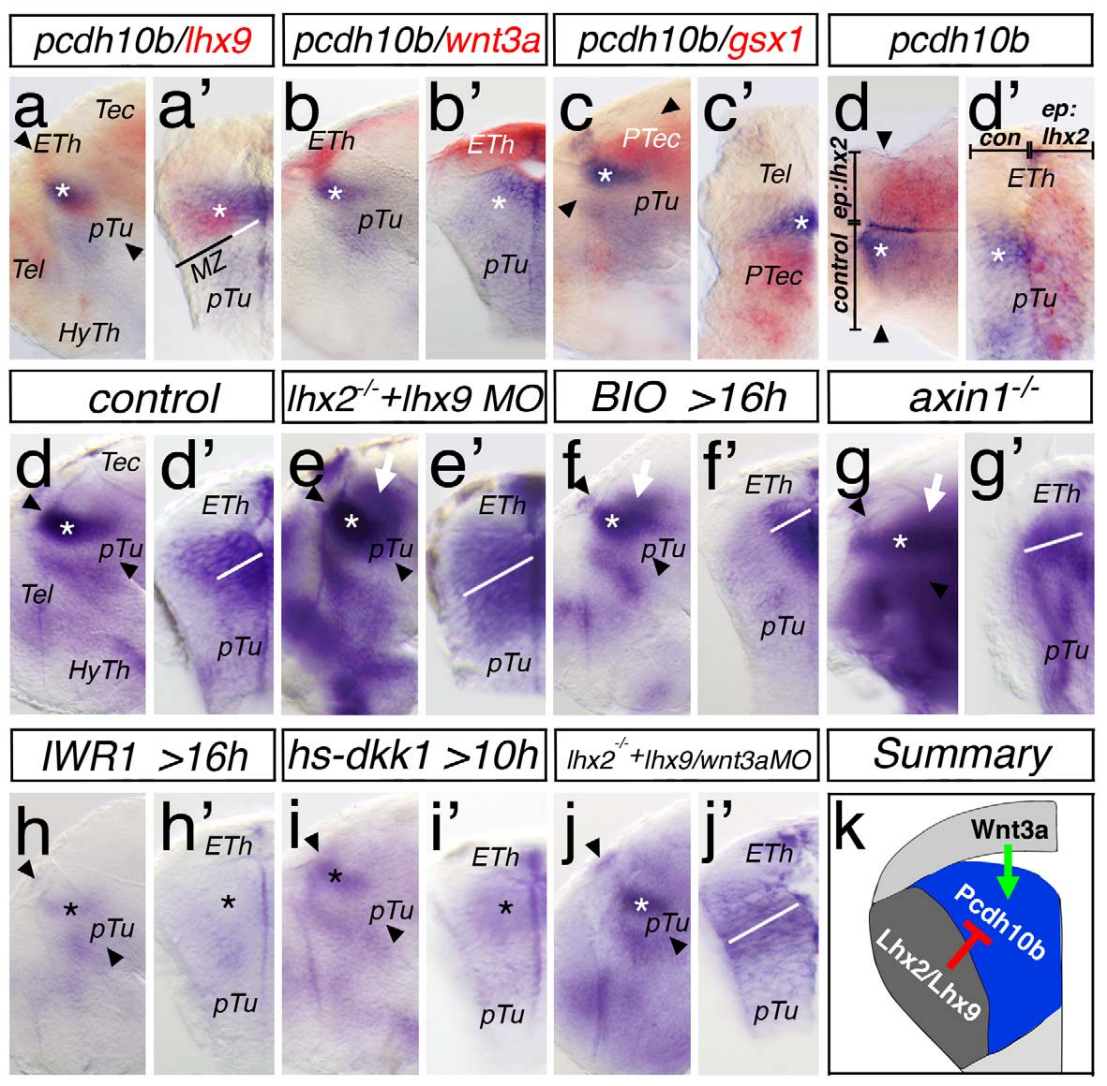
Expression and regulation of *protocadherin10b* in the thalamus. Lateral views and corresponding cross-sections of the left hemisphere of the same embryo at 48 hpf are displayed. Exceptions are a horizontal section in (c′) and dorsal view in (d). Asterisks mark the thalamic territory. *pcdh10b* expression abuts the expression domain of *lhx9* in the mantle zone (MZ, black bar; a, a′). The roof plate marker, *wnt3a*, is adjacently expressed to the *pcdh10b* expression in the thalamus (b, b′). Expression of *pcdh10b* in the thalamus abuts posteriorly the expression domain of *gsx1* and therefore respects the border to the pretecum (c) shown in a dorsal view (c′). Overexpression of *lhx2* DNA via electroporation leads to a unilateral downregulation of *pcdh10b* expression (dorsal view, d; d′). Control embryos show *pcdh10b* expression in the cTh (d, d′). In *lhx2* mutant embryo knocked-down for *lhx9*, *pcdh10b* expression expands into the pretectum (e), and the ventricular expression expands into the MZ (e′, white bar). Treatment of embryos with the Wnt signaling agonist BIO from 16 hpf to 48 hpf leads to an expansion of *pchd10b* expression into the pretectum (f, white arrow), however the expanded VZ is not detectable (f′, white bar). Although the gross morphology is altered, *pcdh10b* expression shows similar broadening in *axin1* mutant embryos (g, g′). Consequently, blocking of Wnt signaling by IWR-1 treatment from 16 hpf to 48 hpf leads to a severe downregulation of *pcdh10b* (h, h′). Embryos with ubiquitous expression of the Wnt inhibitor Dkk1 after heat shock activation at 10 hpf leads to a downregulation of *pcdh10* expression at 48 hpf (i, i′). Knock-down of Wnt3a in the Lhx2/Lhx9-double-deficient embryos leads to a rescue of the expansion into the pretectum (j), however the lateral expansion of the VZ is still detectable (j′). Canonical Wnt signaling—that is, Wnt3a—is required for induction of *pcdh10b* expression in the thalamic ventricular zone, whereas Lhx2/Lhx9 inhibits *pcdh10b* expression in the mantle zone of the cTh (k).

We find that the expression of the cell adhesion molecule, *protcadherin10b* (*pcdh10b*), starts in the cTh during late somitogenesis (Figure S4). At 48 hpf, *pcdh10b* is predominantly expressed in the progenitor layer, non-overlapping with the post-mitotic *lhx2/lhx9* positive neurons (Figure 5a,a′). The expression domain of *pcdh10b* abuts dorsally the expression domain of the epithalamus including the *wnt3a* expression domain (Figure 5b,b′) and posteriorly with the domain of the pretectal marker *gsx1* (Figure 5c,c′). Thus, *pcdh10b* marks specifically caudal thalamic progenitors at 48 hpf.

To investigate the functional interaction between Lhx2/Lhx9 and Pcdh10b, we electroporated *lhx2* DNA unilaterally into the caudal diencephalon. Overexpression of Lhx2 proved to be sufficient to inhibit *pcdh10b* expression in the ventricular zone of the thalamus (16/36; Figure 5c,c′). Furthermore, the thalamic expression domain of *pcdh10b* in *lhx2/lhx9*-deficient embryos expands into the mantle zone of the cTh (17/23, Figures 5d′,e′, S4). This suggests a repressor function of Lhx2 on *pcdh10b* expression. Interestingly, and beyond a direct repressor effect in situ, *pcdh10b* also expanded posteriorly into the normally Lhx2/Lhx9 negative pretectum (Figure 5d,e).

How do we explain this non-autonomous expansion of *pcdh10b* following knock-down of Lhx2/Lhx9? We wondered whether this could be linked to increased Wnt signaling in the diencephalon of Lhx2/Lhx9-depleted embryos. Therefore, we altered canonical Wnt signaling by treating embryos with small molecule effectors of the Wnt signaling pathway such as the activator, BIO (a GSK3ß inhibitor) [47]. To mimic the situation in *lhx2/lhx9* morphant embryos, and to avoid gross malformation due to altered patterning during gastrulation, we started ectopic activation of Wnt signaling at 16 hpf and treated the embryos up to 48 hpf. In treated embryos we see ectopic induction of *axin2* expression at 48 hpf (Figure S4), an expansion of *pcdh10b* expression into the pretectum (30/36; Figure 5f,f′) similar to the outcome from Lhx2/Lhx9 depletion. In BIO treated embryos, the expression pattern of the principal signal of the *MDO*, *shh*, and the patterning marker *pax6a* are unaltered excluding pleomorphic effects of the treatment (Figure S4).

Following these results, we analyzed the expression of *pcdh10b* in embryos carrying a mutation in the Wnt pathway inhibitor Axin1 [48]. Although *axin1* mutants lack most of the telencephalon and the eyes (Figure S4), we find an enlarged expression domain of *pcdh10b* in the cTh at 48 hpf (Figure 5g,g′). Accordingly, we treated embryos with the Wnt signaling antagonist IWR-1 (a tankyrase inhibitor, Figure S4) [49] from 16 hpf to 48 hpf. Inhibition of Wnt signaling exhibits a decrease of *pcdh10b* expression (55/58; Figure 5h,h′). To validate these results, we used a heatshock inducible transgenic fish line to overexpress the canonical Wnt antagonist Dickkopf1, Dkk1 (Figure S4) [50],[51] at 10 hpf. Indeed, we find a similar decrease of *pcdh10b* expression (Figure 5i,i′). This effect is seen before, but not after, endogenous *pcdh10b* induction, suggesting that Wnt signaling is required for induction of *pcdh10b* but not for its maintenance (Figure S4).

To dissect the regulatory contribution of Lhx2/Lhx9 and Wnt signaling to *pcdh10b* expression, we reduced Wnt3a function in Lhx2/Lhx9-deficient embryos (Figure 5j,j′). Interestingly, here we do not find the posterior expansion of the *pcdh10b* expression domain into the pretectum (40/84; Figure 5i). However, we still observe the expansion of *pcdh10b* into the neuronal layer (40/84; Figure 5i′).

In summary, these data suggest that Wnt signaling, most likely by Wnt3a, induces expression of *pcdh10b* in the caudal thalamus and Lhx2/Lhx9 are able to limit *pcdh10b* expression to the progenitor zone (Figure 5k). Furthermore, ectopic upregulation of Wnt signaling is able to induce *pcdh10b* expression also in the ventricular zone of the pretectum.

### Protocadherin10b Mediates Lineage Restriction and Diencephalic Compartition

**Figure 6:**
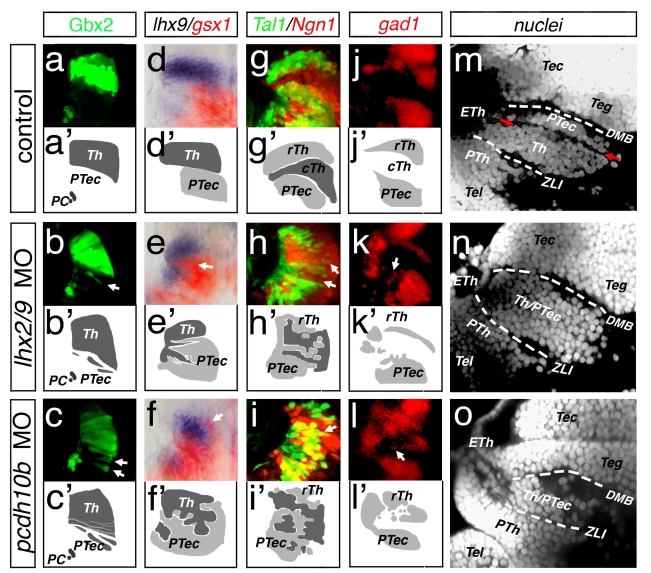
Protocadherin10b is required to maintain integrity of thalamus. Dorsal views of the left hemisphere of embryo at 42 hpf (a–c), 48 hpf (d–i), and 3 dpf (j–l) are displayed. To visualize orientation of the figures, small sketches accompany the experiments showing the thalamus (Th) in dark grey and the rostral thalamus (rTh)/pretectum (PTec) in light grey. At 4 dpf, the anatomy of the caudal forebrain is visualized by a confocal microscopy analysis of ubiquitous nuclei staining by Sytox green (m–o). At 42 hpf, *gbx2*:GFP expression marks the thalamus as well as the position of the diencephalic-mesencephalic border (DMB) by the position of the posterior commissure (PC). Knock-down of Lhx2/Lhx9 leads to the appearance of *gbx2*:GFP positive cells posterior to endogenous expression domain (b, white arrow). In embryos knocked down for Pcdh10b, thalamic *gbx2*:GFP cells appear similarly to (b) in the pretectum (c, white arrows). Analysis of *lhx2/lhx9* morphant embryos and *pcdh10b* morphant embryos by a double ISH approach for *lhx9/gsx1* (d–f). *lhx9* marks the thalamus and *gsx1* the pretectum seen in a dorsal view (d). In *Lhx2/Lhx9* morphant embryos, the expression pattern of *lhx9* and *gsx1* intermingles (e, white arrow) similar to the phenotype observed in *pcdh10b* morphant embryos (f; white arrows). Confocal sectioning of *lhx2/lhx9* double morphant embryos in vivo reveals mixing between Tal1:GFP positive and the neurog1:RFP positive cells in the cTh (g–h′, white arrows). A similar intermingling phenotype is detectable in *pcdh10b* morphant embryos at 48 hpf (i, i′). At 3 dpf, the rTh is marked by *gad1* by a fluorescent ISH (j, j′). After knock-down of Lhx2/Lhx9, *gad1* positive cells can be found in the territory of the cTh (k, white arrows); furthermore, in Pcdh10b-deficient embryos, *gad1* positive can also be found in the cTh (l, l′). Lateral views of the caudal forebrain show three cell nuclei loose border zones: the border between prethalamus and thalamus, the *ZLI* (white dashed lines), the one between the thalamus and the pretectum (red arrows), and the one between pretectum and midbrain *DMB* (white dashed lines). The border zone between the thalamus and the pretectum is not detectable in *lhx2/lhx9* morphant embryos (n). Similarly, this demarcation is also missing in *pcdh10b* morphant embryos (o), whereas the *ZLI* and the *DMB* are not affected. Tec, tectum; Teg, tegmentum.

To study the consequences of altered Pcdh10b levels in the developing caudal forebrain, we analyzed the maintenance of the border zone between thalamus and pretectum in Lhx2/Lhx9 morphant embryos and Pcdh10b-deficient embryos (Figure 6 and Figure S5). We used five different sequential approaches from the onset of neuronal differentiation at 42 hpf to the formation of a mature thalamus at 4 dpf.

Firstly, we analyzed thalamus-specific GFP expression in the Gbx2:GFP transgenic zebrafish line (Figure 6a–c′) [52]. In embryos deficient for Lhx2/Lhx9, we observe that GFP-positive cells in the ventricular zone of the pretectum become detached from the Gbx2:GFP positive thalamus (8/14; Figure 6b,b′, white arrow), suggesting the loss of lineage restriction at the thalamus/prectectum boundary and the spread of thalamic cells into the pretectum. Assuming this to be the case, we next asked if different levels of *pcdh10b* are required to maintain lineage restriction at this border. Therefore, we interfered with Pcdh10b function by using a Morpholino antisense approach for Pcdh10b [53]. In *pcdh10b* morphant embryos we find Gbx2:GFP positive cells ectopically in the pretectal progenitor layer (18/25; Figure 6c,c′, white arrows).

Secondly, we examined the separation of thalamic and pretectal domains by the regional expression of the transcription factors *lhx9* and *gsx1* (Figure 6d–f′). Knock-down of Lhx2/Lhx9 (11/16) or Pcdh10b (46/73) leads to significant intermingling of *lhx9* positive thalamic cells and *gsx1* positive pretectal cells (Figure 6e–f′, white arrows).

Thirdly, considering the relay thalamus being mainly glutamatergic whereas the central pretectum remains mainly GABAergic, we looked at the localization of the ßHLH factors Tal1 and Neurog1. Tal1 marks the inhibitory neurons of the rTh and pretectum, whereas glutamatergic progenitors express Neurog1 [13]. To achieve single-cell resolution, we analyzed the offspring of a Tal1-GFP transgenic line crossed to a Neurog1-RFP transgenic line. We find the specification of ectopic Tal1 positive neurons in the territory of the caudal thalamus in Lhx2/Lhx9 double morphant embryos as well as in Pcdh10b-deficient embryos (Figure 6h–i′).

Fourthly, we analyzed the expression of Gad1, a marker of inhibitory GABAergic neurons by fluorescent ISH at 3 dpf (Figure 6j). In both Lhx2/Lhx9-deficient embryos (4/8) and *pcdh10b* morphant embryos (6/10) *gad1* positive cells are mis-located within the glutamatergic caudal thalamic domain (Figure 6k–l′; white arrows, Figure S5).

Fifthly, we studied the anatomy of the caudal forebrain by analyzing areas of clustered cell nuclei at 4 dpf. In wild type embryos, we observe demarcations between prethalamus and thalamus (the *ZLI*), between the thalamus and the pretectum, and between the pretectum and the midbrain (the *diencephlic-mesencephlic border*; *DMB*) (Figure 6m). The observed anatomical compartition correlates with the described genetic profile of these territories (Figure S5). In *lhx2/lhx9* morphant embryos, the demarcation between the thalamus and pretectum is not detectable, although the *ZLI* and the *DMB* are unaltered (Figure 6n). In *pcdh10b* morphant embryos, we are not able to identify the boundary between pretectum and thalamus (Figure 6o), while the *ZLI* and *DMB* are still visible. We hypothesize that similar adhesive properties in the thalamus and in the pretectum lead to a loss of separation of these brain parts. Thus, we conclude that a Pcdh10b positive thalamus and a Pcdh10b negative pretectum are required to establish a border between these compartments.

## Discussion

### Development of Thalamic Relay Neurons

The molecular mechanisms that control the orderly series of developmental steps leading to mature thalamic neurons are poorly understood. Although numerous transcription factors are specifically expressed in the thalamus [14], only a few have been functionally characterized such as Gbx2, Neurog2, and Her6. Gbx2 knock-out mice show disrupted differentiation of the thalamus by the absence of thalamus-specific post-mitotic neuronal markers *Id4* and *Lef1*, and subsequently lack cortical innervation by thalamic axons [44]. Although Neurog2-knock-out mice show a similarly severe failure in neuronal connectivity to the cortex, the expression of *Lhx2*, *Id2*, and *Gbx2* is unchanged in these mice, suggesting that in the absence of *Neurog2* thalamic neurons are not re-specified at the molecular level [54]. In contrast, Her6 regulates the thalamic neurotransmitter phenotype by repressing *neurog1* function and subsequently the glutamatergic lineage. By contrast, Her6 function is a prerequisite for Ascl1a-positive interneuron development in the GABAergic rostral thalamus [13].

Here, we investigate the function of conserved Lhx2 and Lhx9 expression during thalamic development. Lim-HD genes form paralogs such as Lhx1 and Lhx5, and Lhx2 and Lhx9 [18]. These pairs have been implicated in various aspects of forebrain development. Lhx1/Lhx5 influence Wnt activity by promoting the expression of the Wnt inhibitors sFRPs. This local Lhx-mediated Wnt inhibition is required in the extra embryonic tissue for proper head formation [55] and establishment of the prethalamus [31]. The Apterous group, Lhx2 and Lhx9, is required for multiple steps during neuronal development. Lhx2 is required in mouse for maintenance of cortical identity and to confine the cortical hem, allowing proper hippocampus formation in the adjacent pallium [26],[56]. However, Lhx2 function during diencephalic development is still under debate. Although the Apterous genes are already present in the nervous system of the cephalochordate Amphioxus—that is, AmphiLhx2/9 [57]—and co-expression of Lhx2 and Lhx9 has been documented in the diencephalon of vertebrates, such as zebrafish (here), Xenopus [20],[22], and mouse [21], their function in the thalamus has remained unclear. Recent studies of Lhx2 mutant mice showed no alteration during thalamic neuronal regionalization [58]. Furthermore, the function of Lhx9 has not been described, but the expression pattern suggests a role during forebrain development and possibly in parcellation of the thalamus [21].

Here, we show that single knock-down of Lhx2 or Lhx9 has no diencephalic phenotype with the markers analyzed (Figure S2), comparable to the Lhx2 knock-out mouse, but that simultaneous knock-down of both Lhx2 and Lhx9 leads to stalling of thalamic neurogenesis at the late progenitor stage (Figure 2). Furthermore, the activation of Lhx2 alone is sufficient to compensate for the loss of both Lhx2 and Lhx9 (Figure 3). Our results suggest that Lhx2 is functionally redundant to Lhx9 to ensure proper thalamic development. In contrast to other vertebrates, zebrafish embryos show co-expression of Lhx2 and Lhx9 in the telencephalon until 48 hpf (Figure 1), which could again suggest redundancy [32]. Indeed the pallium is less affected in the *lhx2*−/− mutant fish compared to loss of the neocortex in Lhx2−/− mutant mice [39],[59]. Furthermore, in the *Lhx9* negative nasal placode, the knock-out of *Lhx2* has been shown to lead to a similar neuronal arrest [24],[60].

In the thalamus, Lhx2/Lhx9 may regulate genes that are essential to complete neuronal development, such that neurons do not reach the terminal neuronal stage. In Lhx2/Lhx9 morphant embryos, we find that the expression of *deltaA*, *neurog1*, as well as *pcdh10b* is increased. During neuronal development in fish, Neurog1 has been shown to activate delta genes directly by binding several E-box motives in the delta promoter region [40]. This suggests that in Lhx2/Lhx9 morphant embryos, neuronal progenitor development is arrested at the level of *deltaA*/*neurog1* expression. Consistently, terminal thalamic neuronal markers such as Id2a and Lef1 are absent in Lhx2/Lhx9 morphant embryos. Interestingly, both of these markers have been shown to be activated by Wnt signaling [61],[62]. Although local Wnt activity is upregulated locally in the *lhx2/lhx9* morphant embryos, these target genes are not transcribed, suggesting that Lhx2/Lhx9 thalamic neuronal differentiation is coupled to a second competence phase for Wnt signaling. Also, the late and restricted onset of Lhx2/Lhx9 expression in the thalamus and their requirement for Id2a and Lef1 expression may explain the thalamic neuronal specificity of the Wnt target *lef1*. Thus, we propose that Lhx2/Lhx9 are essential determinants for cells to reach the late stage of thalamic neuronal development.

**Figure 7:**
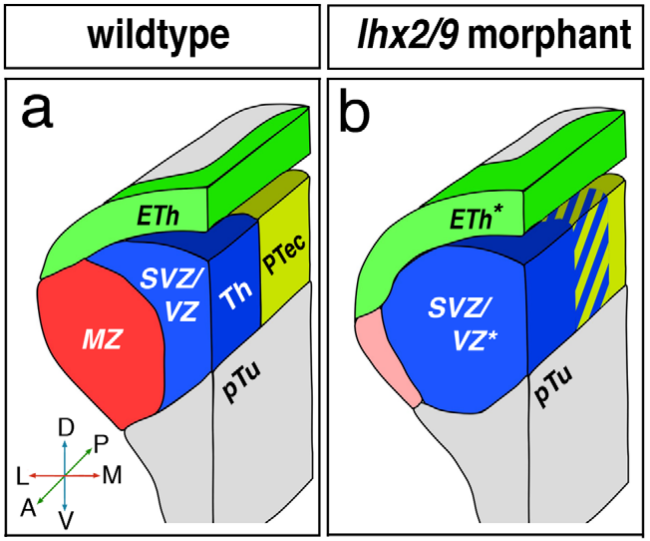
Function of Lhx2/Lhx9 during thalamic neurogenesis and regionalization of the caudal forebrain. The schematic drawing shows a 3-D view of the left hemisphere of the caudal diencephalon and body axis. In Lhx2/Lhx9-deficient embryos, the ventricular and subventricular zone of the thalamus (blue) expands laterally into the mantle zone (red). Furthermore, the Wnt positive epithalamus (green) expands ventrally into the misspecified MZ. Subsequently, upregulation of Wnt signaling in the mid-diencephalon may lead to intermingling of thalamus and pretectum by altered localization of Pcdh10b (yellow/blue stripes).

In the spinal cord, Lim HD factors together with ßHLH factors have been shown to be required for cell cycle exit [63]. The Lim containing factor Isl-1 and Lhx3 together with the ßHLH factors Neurog2 and NeuroM act in a combinatorial manner to directly trigger motor neuron differentiation. In the thalamus, we find a similar process: Lhx2/Lhx9 inhibit the expression of progenitor markers such as *pcdh10b* and activate the expression of postmitotic differentiation markers such as *id2a*, *lef1*, and *elavl3*. Interestingly, proper differentiation of thalamic neurons is required to restrict the *MDO* and dorsal roof plate (Figure 7), a finding that reflects the conversion of neocortex in Lhx2 knock-out mice. Here, the Gdf7 positive cortical hem expands at the expense of the neocortex [23]. This supports the hypothesis that proper neuronal differentiation is required to maintain brain compartments and their borders.

### Wnt Signaling, Pcdh10, and Cell Adhesion

In the mid-diencephalon, the central source of patterning cues is the *MDO*. Here, three different signaling pathways merge: Shh, Fgf, and Wnt [64]. Shh signaling has been shown to induce proneural genes such as Ascl1 in the rostral thalamus and Neurog1 in the caudal thalamus (cTh) [12],[13],[65] and a set of transcription factors assigning specific properties to the developing thalamic cells [14],[21],[66]–[68]. Furthermore, Fgf signaling influences the development of the rTh [69] and parts of cTh, the motor learning area [70]. Interestingly, although the mid-diencephalon expresses a set of canonical and non-canonical Wnt ligands and receptors [27],[28], the function of Wnt signaling is not clear. Wnt signaling seems to be required for mediating thalamic identity in chick embryonic explants [29] and mutation of the Wnt co-receptor Lrp6 leads to a severe reduction of thalamic tissue in mice [30].

Here, we show that Wnt signaling from the *MDO* and the roof plate influence compartition of the caudal diencephalon. The canonical Wnt signaling pathway plays a pivotal role in mediating adhesiveness and the key effector of the Wnt pathway, β-catenin, was initially discovered for its role in cell adhesion [71],[72]: it promotes adhesiveness by binding to the transmembrane, Ca2+-dependent homotypic adhesion molecule cadherin, and links cadherin to the intracellular actin cytoskeleton. Although several classes of molecules are involved in morphogenetic events, cadherins appear to be the major group of adhesion molecules mediating formation of boundaries in the developing CNS [73]. After a phase of ubiquitous expression, cadherins display a very distinct expression pattern in the neural tube [74]. In the developing diencephalon, classical cadherins, such as Chd2, Chd6b, and Chd7, mark presumptive nuclear gray matter structures within developmental compartments [75]. Still, these studies so far are not able to explain the different compartition in the caudal forebrain.

Here, we describe the expression pattern of the non-clustered protocadherin, *pcdh10b*, in the developing diencephalon and show that it marks the ventricular zone of the thalamus at mid-somitogenesis (Figure S5). During somitogenesis, *pcdh10b* modulates cell adhesion and regulates movement of the paraxial mesoderm and somite segmentation [53]. We find that the border of *pcdh10b* expression co-localizes with the border between thalamus and pretectum during diencephalic regionalization (Figure 5). Furthermore, we could link Pcdh10b expression to canonical Wnt signaling. In chick, some hallmarks of lineage restriction for the border between thalamus and pretectum have been observed previously; for example, vimentin and chondroitin sulfate proteglycans are strongly enriched at this border. Similar to the anatomical observation in fish (Figure 6j–l), the chick neural tube shows a morphological ridge where interkinetic movement is disrupted [6]. However, there are conflicting data from direct analyses of cell lineages in the caudal chick forebrain regarding cell compartment borders between thalamus and pretectum [6],[76]. This may be explained by the different stages of analysis. In other vertebrate models, Pcdh10 expression has been reported only at later stages in development, in chicken HH28, and in mouse E15 [77],[78], arguing against a comparable role in these model organisms. However, Pcdh10 together with Pcdh8, 12, 17, 18, and 19 belong to a structurally related subfamily, the non-clustered δ2 protocadherins, and several members indeed show an expression pattern during somitogenesis in mouse [79]. Although we have not carried out direct lineage restriction experiments by tracing small cell clones at the border, we suggest that the thalamic area intermingles with the pretectum when both areas express similar levels of this adhesion molecule (Figure 7). Our data are supported by the fact that *pcdh10b* knock-down or overexpression also lead to a similar phenotype in somite development [53]. Similarly in Gbx2 knock-out mice, thalamus cells start to intermingle with pretectum cells [11]. Interestingly, these authors observe a non-cell autonomous function for this transcription factor and claim a restriction mechanism mediated by an unknown cell adhesion factor. We suggest that, as for Lhx2/Lhx9, Gbx2 is required for the acquisition of proper neuronal identity and the lack of Gbx2 may lead to a similar sequence of events—that is, expansion of the Wnt-positive roof plate and alteration in *pcdh10b* expression. This hypothesis should be tested in the Gbx2 knock-out mouse. Notably, as *pcdh10b* is also expressed in hindbrain rhombomeres [80] its function should be determined during differentiation in this well-studied segmented part of the neural tube; should compartment formation in the caudal forebrain and hindbrain turn out to involve similar molecular effectors, we may reach a unifying mechanism for compartition of the neuraxis—whether it be in the generation of single units (thalamus, pretectum) or iterated modules (rhombomeres).

Thus, we suggest that Lhx2/Lhx9 is required for neurogenesis within the thalamus and is important to maintain longitudinal axis patterning of the CNS also at later stages. Alteration of neurogenesis in a brain part affects the development of the neighboring parts and thus leads to loss of the integrity over compartment boundaries.

## Materials and Methods

### Maintenance of Fish

Breeding zebrafish (*Danio rerio*) were maintained at 28°C on a 14 h light/10 h dark cycle [81]. To prevent pigment formation, embryos were raised in 0.2 mM 1-phenyl-2-thiourea (PTU, Sigma) after 24 hpf. The data we present in this study were acquired from analysis of wild-type zebrafish of KCL (KWT) and of the ITG (AB_2_O_2_) as well as the transgenic zebrafish lines; *tal1:GFP* [82], *hs-dkk1:GFP* [51], *elavl3:GFP* [83], *GA079:RFP* [84], *shh:RFP*, *neurog1:RFP* [41], *gbx2:GFP* [52], and the *belladonna* zebrafish mutant line with a loss of *lhx2* [39] and *masterblind* mutant line carrying a mutation in *axin1* [48]. In *bel/lhx2* mutants, a 22 bp deletion in the third exon causes a frame-shift and therefore a stop codon after the second LIM domain. Embryos were staged [85] and ages are listed as hours post fertilization (hpf).

### Functional Analysis

Transient knock-down of gene expression was performed as described in [13]. We used the following Morpholino-antisense oligomeres (MO, Gene Tools) at a concentration of 0.5 mM: *lhx2* MO (5′-GCT TTT CTC CTA CCG TCT CTG TTT C-3′), *lhx9* MO (5′-AGG TGT TCT GAC CTG CTG GAG CCG T-3′), *wnt3a* MO [86], and *pcdh10b* MO [53]. The injection of MO oligomers was performed into the yolk cell close to blastomeres at one-cell or two-cell stage. For electroporation, embryos were manually dechorionated and mounted laterally in 1.5% low melting-point agarose at 24 hpf. We locally injected 0.5 µg/µl GAP43-GFP DNA solution or 1 µg/µl pCS2+*lhx2* DNA [32] solution in the III brain ventricle. The positive charged anode was positioned on top of the diencephalon, whereas the negative cathode was positioned underneath the diencephalon (Figure 3). For electroporation, we used a platinum/iridium wire with a 0.102 mm diameter (WPI Inc.). During the electroporation procedure the embryo was kept in 1× Ringer as conductive fluid. We used the stimulator CUY21 (Nepa Gene Ltd.) with the following stimulation parameters: 24 V voltage square wave pulse, 4 ms pulse length, 2 ms pulse interval, delivered three times. Settings are based on the published electroporation approaches in [87].

To manipulate Wnt signaling in vivo, we used BIO [47] ((2′Z,3′E)-6-Bromo-indirubin-3′-oxime, TOCRIS Bioscience or IWR-1 [49]; SIGMA) as pharmacological agonist and antagonist of the Wnt signaling pathway. For Wnt signaling analyses, embryos were dechorionated at 16 hpf (15–17-somite stage) and incubated with 4 µM of BIO in 1% DMSO, 40 µM IWR-1 in 0.2% DMSO, or with 1% DMSO only.

### Staining Procedures

Prior to staining, embryos were fixed in 4% paraformaldehyde/PBS at 4°C overnight for further analysis.

Whole-mount mRNA in situ hybridizations (ISH) were performed as described in [88]. Antisense probes were generated from RT-PCR products for the following probes with primer pairs (forward/reverse): *lhx2b*, 5′-AGT GCG TCT CAC GGA AAT CT-3′/5′-GCA TCC ATG ATC GGT CTT CT-3′; *lhx9*, 5′-CGT TGG AGA AAG TGG ACT GG-3′/5′-TGG TGA AGA ATT CCG ATC AA-3′; *sema3d*, 5′-GCT GCA GAA ATC TCC TCG TC-3′/5′-ATT TTG CAC AAG TGG GCA TT-3′; *helt*, 5′-CCA AAA AGC TCG CCT TTA ATC-3′/5′-AAC ATA TTA AGA CGT ATT TAC AGA GCA-3′; *lmx1b.1*, 5′-GAC AAC AGC CGG GAT AAA AA-3′/5′-CCA TCC GAT TGG ACA TTA CC-3′.

The expression pattern and/or antisene RNA probes have been described for *shha* (*formerly known as shh*; [89]), *gsx1* [90], *pax6a* [91], *gbx2* [92], *axin2* [46], *lef1* [93], *wnt3a* [94], *dla* [95], *id2a* [96], *lmx1b.1* [97], *pcdh10b* [53], *gad1* (gad67) [17], and *vglut2.2* [98].

Post-ISH, embryos were re-fixed in 4% paraformaldehyde/PBS at 4°C overnight and transferred to 15% sucrose/PBS and kept for 8 h at 4°C. For embedding, embryos were transferred to a mould filled with 15% sucrose/7.5% gelatine/PBS at 42°C for 10 min. The moulds were kept overnight at 4°C, frozen in liquid nitrogen on the following day, and stored at −80°C until required. Frozen blocks were sectioned coronal with 16 µm thickness on the cryostat.

To reveal neurons that have initiated axogenesis, we used a monoclonal antibody against acetylated tubulin (Sigma, T-6793) in a concentration of 1∶20 as described in [88].

For visualizing cell nuclei, embryos were fixed in 4% paraformaldehyde/PBS at room temperature for 2 h and transferred in 1× PBS. Fixed brains were hemisected and incubated in 25 µM SYTOX nucleic acid stain (Invitrogen) overnight. After washing in 1× PBS brains were mounted laterally for confocal imaging analysis.

### Image Acquisition

Prior to imaging, embryos were deyolked, dissected, and mounted in 70% (v/v) glycerol/PBS on slides with cover slips. Images were taken on Olympus SZX16 microscope equipped with a DP71 digital camera by using the imaging software Cell A. For confocal analysis, embryos were embedded for live imaging in 1.5% low-melting-point agarose (Sigma-Aldrich) dissolved in 1× Ringer's solution containing 0.016% tricaine at 48 hpf. Confocal image stacks were obtained using the Leica TCS SP5 X confocal laser-scanning microscope. We collected a series of optical planes (z-stacks) to reconstruct the imaged area. Rendering the volume in three dimensions provided a view of the image stack at different angles. The step size for the z-stack was usually 1–2 µm and was chosen upon calculation of the theoretical z-resolution of the 40× objective. Images were further processed using Imaris 4.1.3 (Bitplane AG).

## Supporting Information

Figure S1 Expression pattern of *lhx2* and *lhx9* during thalamus development. A double in situ hybridization approach was used for analysis. All embryos were mounted laterally with stages indicated, except (d′) is a dorsal view and (g′) is a cross-section of the left hemisphere. *lhx9* reveals an onset of expression in the thalamus (Th) at 22 hpf (a, asterisk), limited anteriorly by *shh*, a marker of the *MDO* and posteriorly by *gsx1*, a marker of the pretectum (PTec). At 28 hpf, *lhx2* shows an onset of expression in the thalamus (b, asterisk). Within the thalamus, at 28 hpf *helt* marks the rostral thalamus (rTh) and the pretectum (c), however the *lhx9* expression domain shows no overlap with the *helt* domain. The epithalamus is marked by the Wnt ligand, *wnt3a*, and the expression of the Wnt reporter 7×TCF-siam:GFP (d). The dorsal view reveals lateral a stronger expression of *gfp*-mRNA in comparison to the *wnt3a* pattern (d′). At 48 hpf, *lhx2* and *lhx9* show specific expression patterns in the telencephalon (Tel), thalamus (asterisk), and ventral to the tectum (Tec), indicated by the overlapping expression domain of *pax6a*, marking the alar plate of the forebrain during development (e, f). *axin2* expression in the thalamus co-localizes with the *lhx9* expression. (g, g′). *vglut2.2*, a marker of *glutamatergic neurons* in the relay thalamus (cTh), shows an overlapping expression domain with *lhx9* (h). Both genes, *lhx2* and *lhx9*, mark the thalamus at 3 dpf (i). ETh, epithalamus; HyTh, hypothalamus; *MDO*, mid-diencephalic-organizer; PTec, pretectum; RP, roof plate; rTh, rostral thalamus; Tec, tectum ; Tel, telencephalon.
(TIF)

Figure S2 Efficient knock-down of *lhx2* and *lhx9* during forebrain development. To validate the efficiency of the *lhx2* and *lhx9* splice-site Morpholino-antisense oligomere approach, we isolated cDNA from injected and non-injected embryos at 48 hpf. A PCR approach, with primers flanking exon1 and exon2 of *lhx2*, demonstrates a suppression of the splicing event of intron1 (1.5 kb) in five individual embryos injected with *lhx2* MO (emb1–5) compared to a control embryo (con, 221 bp) (a). A similar effect is demonstrated in injected *lhx9* MO embryos 1–4 (emb1–4; b), which display a non-splicing event of intron1 (993 bp), compared to control embryos (con, 231 bp) (b). An antibody against acetylated tubulin shows midline crossing axons anterior (AC, anterior commissure) and posterior (POC, post-optic commissure) in the telencephalon (c). In *lhx2/lhx9* double morphant embryos, both commissures do not cross the midline (arrow, d). Single in situ hybridizations of embryos at 48 hpf are displayed by a lateral view (e–l). Knock-down of Lhx2 and Lhx9 leads to a decrease of *sema3d* expression in postoptic commissure (POC, arrow; e, f). The morphant analysis of single knock-down, either *lhx2* or *lhx9*, shows that *lef1* expression is unaltered in the thalamus (h, j), compared to the control embryos (g, i, k). In the *lhx2* mutant embryos, *lef1* expression in the thalamus shows a weak alteration (l). HyTh, hypothalamus; *MDO*, mid-diencephalic-organizer; pTu, posterior tuberculum; RP, roof plate; Tec, tectum; Tel, telencephalon.
(TIF)

Figure S3 *lhx2/lhx9* morphant embryos show defect in thalamic neuron differentiation. A single in situ hybridization approach was used for analysis and all embryos were mounted laterally except in (I′, j′) showing cross-section of left hemispheres. Stages are indicated. In Lhx2/Lhx9-deficient embryos, *lef1* expression in the thalamus (asterisk) is unaltered at 24 hpf but down-regulated at 3 dpf (a–d). Similarly, the Wnt target gene *axin2* shows no alteration in Lhx2/Lhx9-deficient embryos at 20 hpf (e, f), however at 3 dfp an up-regulation can be detected in the mid-diencephalon (g, h). In control MO embryos, *wnt1* is expressed at 48 hpf in the roof plate (RP) and *lhx2/lhx9* morphant embryos display an expansion of the *wnt1* expression domain into the thalamic territory (i–j′). In contrast *tcf7l2* shows no alteration in the expression pattern at the same stage in the caudal forebrain. HyTh, hypothalamus; pTu, posterior tuberculum; RP, roof plate; Tec, tectum; Tel, telencephalon.
(TIF)

Figure S4 The thalamic expression of protocadherin10b and its regulation. All embryos are analyzed by a single in situ hybridization approach and mounted laterally, with stages indicated, except (c′) shows a cross-section and the left hemisphere is displayed. In the thalamus (asterisk) *pcdh10b* reveals an onset of expression in segmentation phase (18 hpf), which increases during development (a, b). Knock-down of Lhx2/Lhx9 leads to an expansion of *pcdh10b* expression into the pretectum (pTec, c), as well as of the ventricular zone (VZ, white bar, c′). Black arrowheads indicate the plane of a cross-section. To validate the efficiency of pharmacological treatment with the Wnt signaling agonist BIO or antagonist IWR-1, we also analyzed under the same conditions the Wnt target gene *axin2*. Treatment with the Wnt signaling agonist BIO demonstrates an up-regulation of *axin2*, displayed lateral (d, e). *Axin2* expression is upregulated in *axin1* mutant embryo *masterblind* (*mbl*, f). The treatment of embryos with the Wnt signaling antagonist IWR-1 leads to a loss of *axin2* in the diencephalon (g, h). We find a similar reduction of *axin2* expression in embryos expressing Dkk1 post-heat-shock at 16 h (i). Treatment of embryos with the Wnt agonist BIO has no effect in the expression of shh or pax6a in the forebrain (j–m). In contrast, embryos treated with the antagonist IWR-1 after endogenous *pcdh10b* induction between 24 hpf and 48 hpf show no change in *pcdh10b* expression pattern. HyTh, hypothalamus; pTec, pretectum; pTu, posterior tuberculum; RP, roof plate; Tec, tectum; Tel, telencephalon.
(TIF)

Figure S5 Mapping of the diencephalon in larval stage via SYTOX nuclei staining. Analyses at 48 hpf, lateral view (a, b, c) and dorsal sections of left hemispheres (d–f′) are shown. Lhx9 marks the thalamus (a) and gsx1 the pretectum (a). In *lhx2/lhx9* and *pcdh10b* morphant embryos, the expression domains overlap. A similar intermingling of expression domains is visible in embryos stained for *vglut2.2* and *gad1* (d–f′). Embryos have been analyzed at 4 dpf by a confocal microscopy analysis of ubiquitous nuclei staining by Sytox (g–j″). The analyzed section of the lateral view except dorsal view (h′) is indicated by a schematic drawing (insert). A sytox staining in green reveals structures of the forebrain and midbrain (g). To confirm the position of the thalamus, we analyzed the *shh*:RFP transgenic line, marking the *MDO* anterior to the thalamus (Th, b, b′). The position of the thalamus and pretectum (PTec) was mapped in the *neurog1*:RFP transgenic line (i–i″). To distinguish between the caudal thalamus (cTh) and pretectum, we also analyzed the *tal1*:GFP transgenic line. It labels GABAergic neurons of the rostral thalamus (rTh) and pretectum and therefore identifies the cell nuclei loose border zone between thalamus and pretectum (j–j″). ETh, epithalamus; HyTh, hypothalamus; *MDO*, mid-diencephalic-organizer; PC, posterior commissure; PG, preglomerular complex; PTec, pretectum; PTh, prethalamus; pTu, posterior tuberculum; RP, roof plate; rTh, rostral thalamus; Tec, tectum; Tel, telencephalon; Th, thalamus.
(TIF)

## Abbreviations

cTh: caudal thalamus
*DMB*: *diencephalic-mesencephalic border*
Eth: embryonic epithalamus
hpf: hours post fertilization
IGL: intergeniculate leaflet
ISH: in situ hybridizations
Lhx: LIM homeobox
*MDO*: *mid-diencephalic organizer*
MZ: mantle zone
rTh: rostral thalamus
SVZ: subventricular zone
vLGN: ventral lateral geniculate
VZ: ventricular proliferation zone
*ZLI*: z*ona limitans intrathalamica*
