# Supplementary figures and images for: Lhx2 and Lhx9 Determine Neuronal Differentiation and Compartition in the Caudal Forebrain by Regulating Wnt Signaling
Source: PLoS Biol. 2011 Dec 13;9(12):e1001218. doi: 10.1371/journal.pbio.1001218 (PMC3236734; doi:10.1371/journal.pbio.1001218)

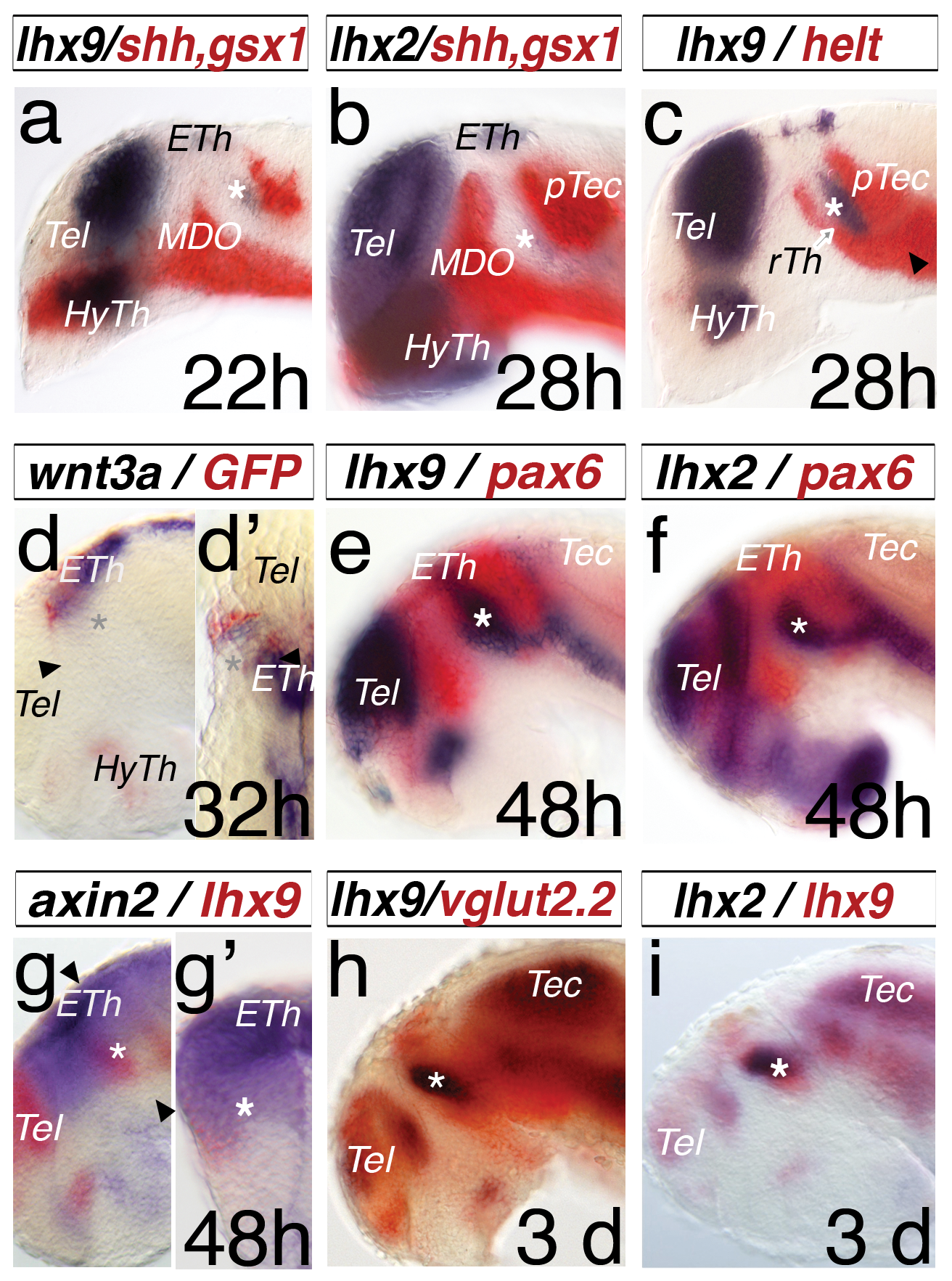

Supplement: Figure S1 — Expression pattern of lhx2 and lhx9 during thalamus development. A double in situ hybridization approach was used for analysis. All embryos were mounted laterally with stages indicated, except (d′) is a dorsal view and (g′) is a cross-section of the left hemisphere. lhx9 reveals an onset of expression in the thalamus (Th) at 22 hpf (a, asterisk), limited anteriorly by shh, a marker of the MDO and posteriorly by gsx1, a marker of the pretectum (PTec). At 28 hpf, lhx2 shows an onset of expression in the thalamus (b, asterisk). Within the thalamus, at 28 hpf helt marks the rostral thalamus (rTh) and the pretectum (c), however the lhx9 expression domain shows no overlap with the helt domain. The epithalamus is marked by the Wnt ligand, wnt3a, and the expression of the Wnt reporter 7×TCF-siam:GFP (d). The dorsal view reveals lateral a stronger expression of gfp-mRNA in comparison to the wnt3a pattern (d′). At 48 hpf, lhx2 and lhx9 show specific expression patterns in the telencephalon (Tel), thalamus (asterisk), and ventral to the tectum (Tec), indicated by the overlapping expression domain of pax6a, marking the alar plate of the forebrain during development (e, f). axin2 expression in the thalamus co-localizes with the lhx9 expression. (g, g′). vglut2.2, a marker of glutamatergic neurons in the relay thalamus (cTh), shows an overlapping expression domain with lhx9 (h). Both genes, lhx2 and lhx9, mark the thalamus at 3 dpf (i). ETh, epithalamus; HyTh, hypothalamus; MDO, mid-diencephalic-organizer; PTec, pretectum; RP, roof plate; rTh, rostral thalamus; Tec, tectum ; Tel, telencephalon. (TIF) [file pbio.1001218.s001.tif]

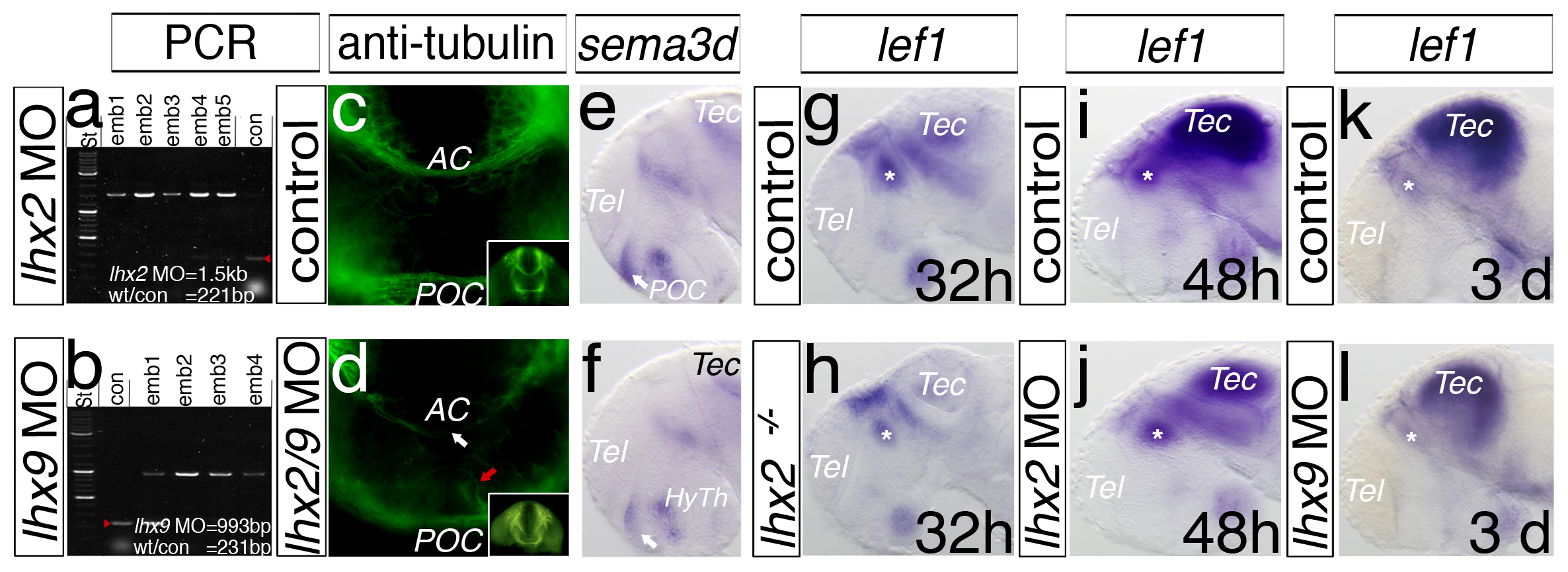

Supplement: Figure S2 — Efficient knock-down of lhx2 and lhx9 during forebrain development. To validate the efficiency of the lhx2 and lhx9 splice-site Morpholino-antisense oligomere approach, we isolated cDNA from injected and non-injected embryos at 48 hpf. A PCR approach, with primers flanking exon1 and exon2 of lhx2, demonstrates a suppression of the splicing event of intron1 (1.5 kb) in five individual embryos injected with lhx2 MO (emb1–5) compared to a control embryo (con, 221 bp) (a). A similar effect is demonstrated in injected lhx9 MO embryos 1–4 (emb1–4; b), which display a non-splicing event of intron1 (993 bp), compared to control embryos (con, 231 bp) (b). An antibody against acetylated tubulin shows midline crossing axons anterior (AC, anterior commissure) and posterior (POC, post-optic commissure) in the telencephalon (c). In lhx2/lhx9 double morphant embryos, both commissures do not cross the midline (arrow, d). Single in situ hybridizations of embryos at 48 hpf are displayed by a lateral view (e–l). Knock-down of Lhx2 and Lhx9 leads to a decrease of sema3d expression in postoptic commissure (POC, arrow; e, f). The morphant analysis of single knock-down, either lhx2 or lhx9, shows that lef1 expression is unaltered in the thalamus (h, j), compared to the control embryos (g, i, k). In the lhx2 mutant embryos, lef1 expression in the thalamus shows a weak alteration (l). HyTh, hypothalamus; MDO, mid-diencephalic-organizer; pTu, posterior tuberculum; RP, roof plate; Tec, tectum; Tel, telencephalon. (TIF) [file pbio.1001218.s002.tif]

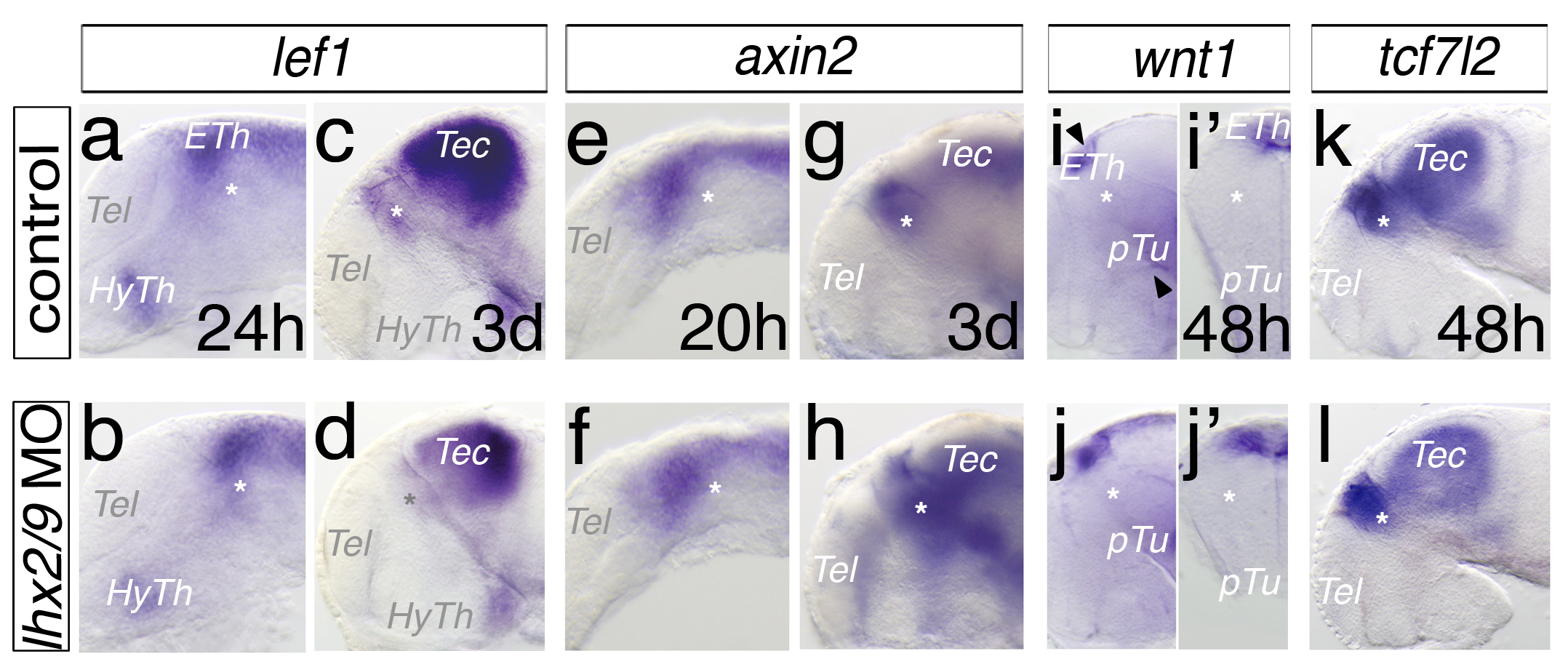

Supplement: Figure S3 — lhx2/lhx9 morphant embryos show defect in thalamic neuron differentiation. A single in situ hybridization approach was used for analysis and all embryos were mounted laterally except in (I′, j′) showing cross-section of left hemispheres. Stages are indicated. In Lhx2/Lhx9-deficient embryos, lef1 expression in the thalamus (asterisk) is unaltered at 24 hpf but down-regulated at 3 dpf (a–d). Similarly, the Wnt target gene axin2 shows no alteration in Lhx2/Lhx9-deficient embryos at 20 hpf (e, f), however at 3 dfp an up-regulation can be detected in the mid-diencephalon (g, h). In control MO embryos, wnt1 is expressed at 48 hpf in the roof plate (RP) and lhx2/lhx9 morphant embryos display an expansion of the wnt1 expression domain into the thalamic territory (i–j′). In contrast tcf7l2 shows no alteration in the expression pattern at the same stage in the caudal forebrain. HyTh, hypothalamus; pTu, posterior tuberculum; RP, roof plate; Tec, tectum; Tel, telencephalon. (TIF) [file pbio.1001218.s003.tif]

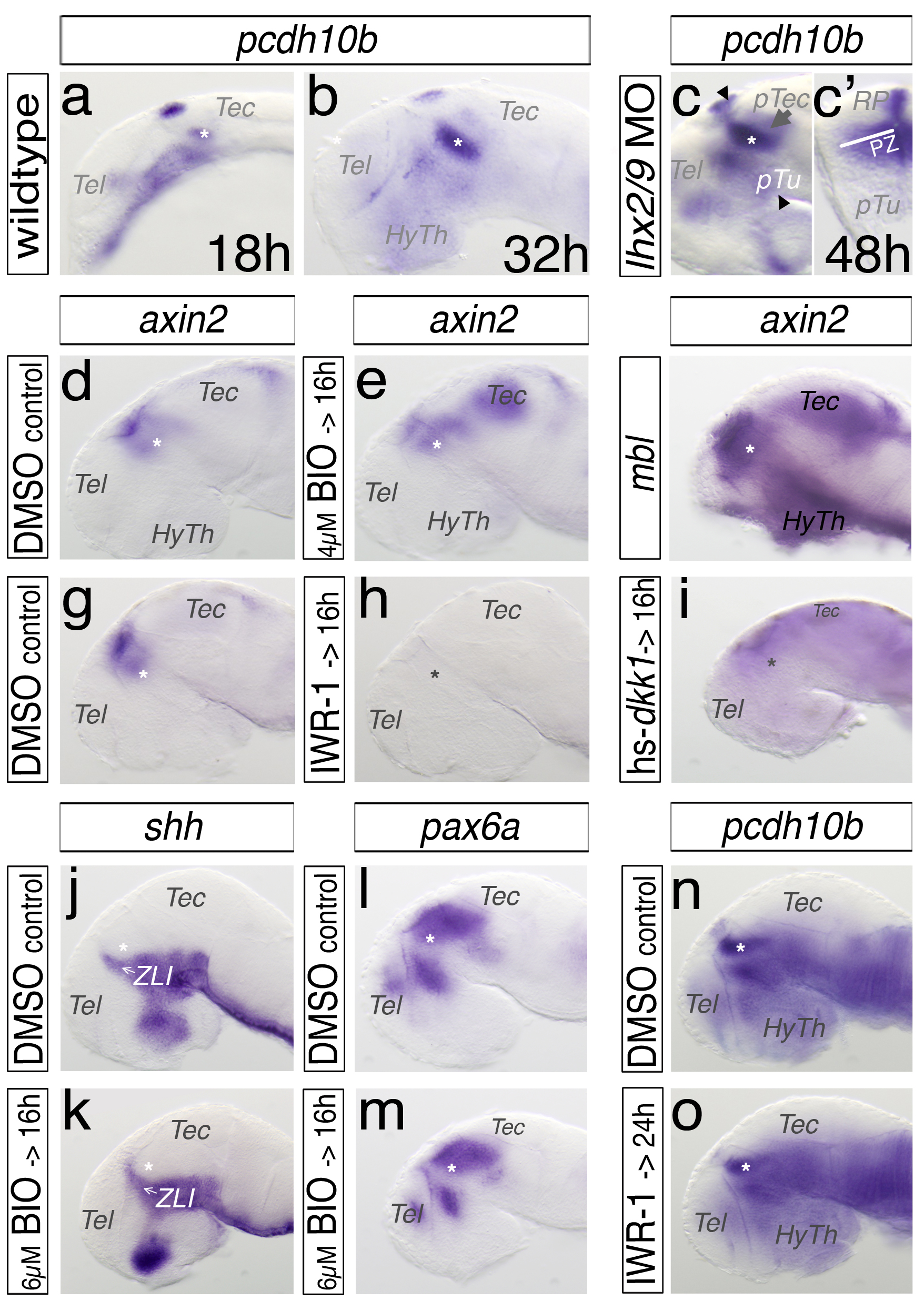

Supplement: Figure S4 — The thalamic expression of protocadherin10b and its regulation. All embryos are analyzed by a single in situ hybridization approach and mounted laterally, with stages indicated, except (c′) shows a cross-section and the left hemisphere is displayed. In the thalamus (asterisk) pcdh10b reveals an onset of expression in segmentation phase (18 hpf), which increases during development (a, b). Knock-down of Lhx2/Lhx9 leads to an expansion of pcdh10b expression into the pretectum (pTec, c), as well as of the ventricular zone (VZ, white bar, c′). Black arrowheads indicate the plane of a cross-section. To validate the efficiency of pharmacological treatment with the Wnt signaling agonist BIO or antagonist IWR-1, we also analyzed under the same conditions the Wnt target gene axin2. Treatment with the Wnt signaling agonist BIO demonstrates an up-regulation of axin2, displayed lateral (d, e). Axin2 expression is upregulated in axin1 mutant embryo masterblind (mbl, f). The treatment of embryos with the Wnt signaling antagonist IWR-1 leads to a loss of axin2 in the diencephalon (g, h). We find a similar reduction of axin2 expression in embryos expressing Dkk1 post-heat-shock at 16 h (i). Treatment of embryos with the Wnt agonist BIO has no effect in the expression of shh or pax6a in the forebrain (j–m). In contrast, embryos treated with the antagonist IWR-1 after endogenous pcdh10b induction between 24 hpf and 48 hpf show no change in pcdh10b expression pattern. HyTh, hypothalamus; pTec, pretectum; pTu, posterior tuberculum; RP, roof plate; Tec, tectum; Tel, telencephalon. (TIF) [file pbio.1001218.s004.tif]

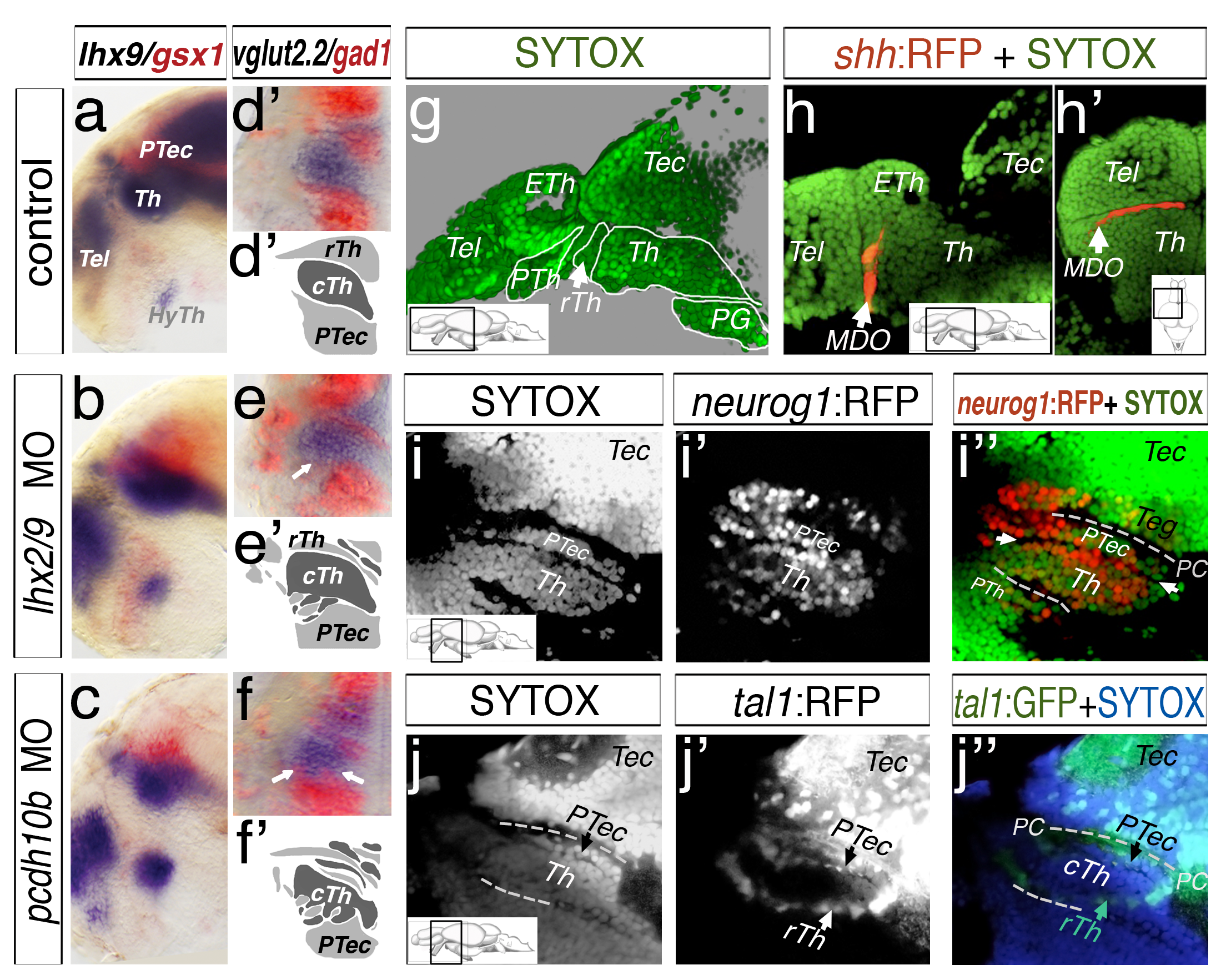

Supplement: Figure S5 — Mapping of the diencephalon in larval stage via SYTOX nuclei staining. Analyses at 48 hpf, lateral view (a, b, c) and dorsal sections of left hemispheres (d–f′) are shown. Lhx9 marks the thalamus (a) and gsx1 the pretectum (a). In lhx2/lhx9 and pcdh10b morphant embryos, the expression domains overlap. A similar intermingling of expression domains is visible in embryos stained for vglut2.2 and gad1 (d–f′). Embryos have been analyzed at 4 dpf by a confocal microscopy analysis of ubiquitous nuclei staining by Sytox (g–j″). The analyzed section of the lateral view except dorsal view (h′) is indicated by a schematic drawing (insert). A sytox staining in green reveals structures of the forebrain and midbrain (g). To confirm the position of the thalamus, we analyzed the shh:RFP transgenic line, marking the MDO anterior to the thalamus (Th, b, b′). The position of the thalamus and pretectum (PTec) was mapped in the neurog1:RFP transgenic line (i–i″). To distinguish between the caudal thalamus (cTh) and pretectum, we also analyzed the tal1:GFP transgenic line. It labels GABAergic neurons of the rostral thalamus (rTh) and pretectum and therefore identifies the cell nuclei loose border zone between thalamus and pretectum (j–j″). ETh, epithalamus; HyTh, hypothalamus; MDO, mid-diencephalic-organizer; PC, posterior commissure; PG, preglomerular complex; PTec, pretectum; PTh, prethalamus; pTu, posterior tuberculum; RP, roof plate; rTh, rostral thalamus; Tec, tectum; Tel, telencephalon; Th, thalamus. (TIF) [file pbio.1001218.s005.tif]
